# Achieving Equitable Access to Obstetric Devices Through Innovation, Improvisation and Off‐Label Use

**DOI:** 10.1111/1471-0528.70058

**Published:** 2025-10-14

**Authors:** G. Justus Hofmeyr, Mandisa Singata‐Madliki, Sara Della Ripa, Andrew D. Weeks

**Affiliations:** ^1^ Effective Care Research Unit University of the Witwatersrand and Walter Sisulu University East London South Africa; ^2^ Department of Obstetrics and Gynaecology University of Botswana Gaborone Botswana; ^3^ ArcHealth Foundation Hinesburg Vermont USA; ^4^ Sanyu Research Unit, Department of Women's and Children's Health University of Liverpool Liverpool UK

**Keywords:** BabySaver, frugal innovation, induction of labour, MaternaWell, medical devices, off label medical devices, postpartum haemorrhage, suction tube uterine tamponade, uterine balloon tamponade, World Health Organisation

## Abstract

The global impact of life‐saving medical devices is directly related to their availability. Access may be limited by cost, availability, or lack of information regarding effectiveness and safety. Addressing the inequity in access requires concerted effort from device developers, the research community, global agencies and professional organisations. We discuss, with examples, three strategies to promote equity: low‐cost, purpose‐built innovation, improvisation and off‐label use. First, developing simple, safe and low‐cost innovative devices can be an effective way of increasing global access. For example, the BabySaver Kit facilitates intact‐cord neonatal bedside resuscitation. Re‐usability is an important design feature for both cost and environment, exemplified by the MaternaWell tray for blood loss monitoring after birth. A second strategy is improvisation using commonly available hospital items. This can extend device availability into settings where purpose‐designed devices are unavailable or unaffordable. Examples include the use of condoms or glove balloons for uterine balloon tamponade (UBT) to treat postpartum haemorrhage (PPH), elastic catheters for uterine tourniquet, and plastic tubing for posterior axilla sling traction in shoulder dystocia. However, the lack of systematically developed evidence and governance approvals can lead to wide variation in training, technique, and device specifications. Finally, some of these quality issues are addressed by using approved medical devices ‘off‐label.’ However, they can have similar problems of variation in technique and depend on the uncoordinated efforts of researchers and clinicians to generate an evidence base. Examples include the Foley catheter for labour induction and the Levin stomach tube for suction tube uterine tamponade for PPH. WHO has pathways to facilitate global access to important public health device innovations. Global agencies and professional organisations also have a major role to play in providing co‐ordination, platforms for data sharing, practice guidelines, instructions for use on off‐label devices and robust data on their safety and effectiveness.

## 1 Introduction

From a public health perspective, the global impact of health innovations depends not only on their effectiveness but, to a major extent, on access to them (coverage). Yet access is shaped by multiple factors, including distribution, cost, regulatory requirements and promotion by influencers such as commercial companies, global agencies and professional organisations. Because access often relates to resource availability, significant global inequities exist, with health innovations frequently taking decades to reach meaningful scale in LMICs [1]. To overcome these barriers, global health professionals are exploring solutions such as expanding manufacturing, new businesses, market‐shaping efforts, innovative incentive structures, and streamlined regulatory models. Such successes have been demonstrated in the example of monoclonal antibody technology availability in low‐resource settings [2].

When medical innovations flow from the Global North to the Global South, efforts to overcome cost barriers often focus on developing low‐cost alternatives. However, these versions may or may not be contextual and still require regulatory registration, governmental approval, integration into guidelines, local procurement and distribution. These challenges have driven the development of innovative, contextual and low‐cost solutions within low‐resource settings. There is a particularly strong call for these actions in the global surgery field: ‘The Global Surgical community […] should actively search for and validate the efficacy of innovations introduced in LMICs, push for the acceptance of these methods and campaign with ferocity for the financial gains of innovations from LMICs to benefit those who have invented and pioneered them.’ [3].

Meanwhile, achieving equitable access in underserved regions often relies on the improvisation and off‐label use of existing local materials and devices. This is a practice that plays an important and complex role, offering potential benefits but also carrying limitations and risks. The primary advantage of improvisation and off‐label use lies in their immediate and widespread availability. However, limitations include the absence of education surrounding the indication by commercial companies or global influencers, as well as the lack of formal instructions for use. There are also clinical and medico‐legal [4] risks stemming from limited robust data and the absence of safety checks typically built into regulatory processes.

In the absence of regulatory registrations and industry accountability to ensure the safe use of purpose‐built products, global agencies and professional organisations have stepped in to play an important role in defining evidence‐based recommendations for several improvised devices and off‐label obstetric products.

In this paper we will discuss low‐cost, purpose‐built innovation, improvisation and off‐label use of obstetric health technologies (Table 1), with examples that illustrate the potential of these practices to impact global health inequity.

**TABLE 1:** features of three strategies to extend access to medical devices.

	Low‐cost innovation	Improvisation	Off‐label use
Definition	Contextualised, low‐cost, regulatory‐registered medical technology, often re‐usable	Utilisation of commonly available materials (not necessarily registered medical products) for medical applications, often requiring assembly step(s)	Use of regulatory‐registered medical products (medications, devices, etc.) outside of their approved indications or populations
Examples	MaternaWell Tray; BabySaver neonatal resuscitation platform	Condom or glove uterine balloons; rubber catheters for uterine tourniquet; plastic catheters for shoulder dystocia axilla sling traction	Foley catheter for labour induction; various catheters for suction uterine tamponade to treat PPH
Benefits	Affordability	Affordability, immediate wide availability: no need for registration or new distribution pathway	
Risks	Unanticipated adverse effects, supply chain issues, can be commercially unsustainable	Lack of safety testing and standardisation; variation in clinical use	
Barriers	Registration and distribution expenses make it difficult to keep product costs low	Lack of manufacturer instructions for use; lack of awareness and training programmes; user concern for medico‐legal exposure	
Policy needs	Global agency support and recommendation	Global agency coordination of research and guideline development, data sharing and evidence dissemination	

## 2 Low Cost Innovations

The costs of life‐saving technologies can be reduced through innovative approaches that prioritise high‐quality, cost‐effective solutions tailored to resource‐limited settings. Reusability is also important as it extends the lifespan and cost efficiency of medical devices. This can be achieved through durable design, modular components or improved sterilisation methods. However, this approach creates inherent commercial barriers as simplicity and reusability mean that profit margins are small and cannot support the levels of advertising and promotion that typically accompany the launch of a medical device. Device simplicity is also commercially challenging due to the need to protect intellectual property and create financial incentives to compete successfully in the medical device industry. This is often achieved by differentiating products to secure patents, which serve as valuable assets for investors and justify higher product costs.

Re‐usability, while ecologically sound, cost‐efficient and more sustainable than disposables, is less popular with health workers who have become accustomed to a ‘use‐and‐discard’ culture. One possible solution may be that the massive financial benefit to manufacturers of disposable over re‐usable devices be offset by realistic taxation on the environmental cost of disposables.

### 2.1 MaternaWell Tray

The MaternaWell Tray is a reusable device for blood loss monitoring after birth which, compared with the standard disposable drapes, reduces costs and carbon emissions while eliminating the logistical barrier of maintaining supply chains for disposables. Barriers to uptake include a lack of research data, as disposable drapes have been standard for postpartum haemorrhage (PPH) research for decades, and the reluctance of health care providers in settings with prior access to the convenience of disposables to change entrenched behaviours. A systematic review has indicated that the amount of RCT evidence on the MaternaWell Tray is very small in comparison to disposable calibrated drapes [5]. On the other hand, anecdotal user experience in Sierra Leone has been positive: ‘The nurses and midwives are typically very hesitant to diagnose and write down a PPH but I think this objective overflow of blood from one well to the other well makes it much easier for them to ‐ it becomes a yes/no issue for diagnosis of PPH which is really necessary when working with lower cadre staff. We love it!’ [6] The perspectives of patients are critical to the issue of implementation. Two patient surveys found high rates of acceptability of the MaternaWell tray [7, 8].

### 2.2 The BabySaver Neonatal Resuscitation Platform

Despite evidence of up to 69% reduction in neonatal mortality [9], delayed cord clamping for preterm neonates is seldom practised in low‐resource settings. The BabySaver is a simple, re‐usable plastic tray with instructions to facilitate neonatal resuscitation at the beside [10]. Currently in commercial development, it will be a fraction of the cost of electronic resuscitation stations and does not require electricity. Apart from its practical use, it may act as a catalyst for behaviour change, which is the most refractory barrier to the implementation of life‐saving delayed cord clamping.

There are many other examples, for example the LifeWrap Non‐Pneumatic Anti‐Shock Garment for first aid management of shock and PPH [11]; the Ellavi balloon for intrauterine balloon tamponade [12], and the CRADLE Vital Signs Alert device [13]. There are also companies that specialise in the development, marketing, and distribution of high‐quality low‐cost devices (e.g., Laerdal Global Health (https://laerdalglobalhealth.com) and Maternova (https://maternova.net)).

## 3 Improvisation

Improvisation involves the use of commonly available hospital materials such as gloves, condoms, or tubes for medical applications. Whilst usually developed out of desperation in the absence of alternatives, some have now entered common usage.

### 3.1 Uterine Balloon Tamponade Devices (UBT) for PPH

UBT is recommended by most international guidelines [14], and the condom‐catheter improvisation has been promoted in low‐income settings by several academic institutions and international funding agencies [15, 16]. Although its use has been supported by observational data [17], two randomised trials have found worse outcomes with the condom‐catheter UBT when compared with standard care [18, 19, 20]. A systematic review found unclear effects of UBT [21] and the World Health Organization (WHO) has qualified its recommendation [22].

### 3.2 Shoulder Dystocia

Another improvised innovation that has seen global uptake is the posterior axilla sling traction for shoulder dystocia [23]. In this technique, any simple plastic tube (such as the infant suction tube available in most labour wards) is put under the fetal arm to assist with delivery [24]. However, the risk of improvised use is illustrated by a report of neonatal trauma when an abrasive elastic catheter was used instead of a smooth, inelastic plastic tube [25].

### 3.3 Uterine Tourniquet and Transvaginal Uterine Artery Clamping for PPH

Uterine tourniquet using any elastic catheter such as the Foley catheter has been widely reported to reduce uterine bleeding associated with myomectomy and PPH [26].

Similarly, transvaginal clamping of the uterine arteries is an intuitively direct and simple way of reducing blood loss during PPH, with recent evidence of effectiveness from a small randomised trial [27]. However, these potentially life‐saving interventions are seldom used as they are, to our knowledge, not included in global or national guidelines such as NICE, and clinicians are often unaware of them.

## 4 Off‐Label Use of Available Low‐Cost Devices

Off‐label use of accessible technologies may offer a viable and equitable alternative. Although this article addresses medical devices, the benefits and, importantly, the risks of ‘off‐label’ drug use are well illustrated by examining the original ‘off‐label’ use of misoprostol in obstetrics by clinical trial and error, investigator‐initiated research then guideline development [28, 29]. Whilst licensed products are now available, the current widespread availability of this affordable prostaglandin analogue has been achieved at the cost of harm such as many more widespread uterine ruptures from ‘trial and error’ clinical overdosage for labour induction than would have happened during limited dose‐finding studies [30]. The dosage recommended for PPH may still be unnecessarily high [31].

Given the complexity and expense of developing novel devices, many clinicians use easily available devices off‐label to perform the necessary tasks, in much the same way that misoprostol was adapted. Pathways are needed to ensure that the inevitable off‐label use of medical devices receives similar attention as commercial products to promote safety and access. Below, we will describe several off‐label maternity devices to illustrate their development and discuss the current advances and controversies over devices to treat PPH.

### 4.1 Labour Induction

An uncontroversial example of successful off‐label use is the Foley urinary catheter use for mechanical labour induction [32]. Almost all major trials of balloon cervical ripening in both high‐ and low‐middle income countries have used the Foley catheter balloons off‐label. The purpose‐designed double balloon device has not been found to be more effective or safe than the Foley catheter balloon and is associated with more pain during insertion and labour induction [33]. The widespread availability of Foley catheters makes the global impact of this important technology vastly greater and more equitable than one relying on procurement of a purpose‐designed device. This off‐label use has recently been extended by using 3 balloons side by side when the cervix is too dilated to retain a single balloon [34].

### 4.2 Suction Tube Uterine Tamponade (STUT) for Refractory PPH

An alternative approach to uterine balloon tamponade is the use of negative intrauterine pressure to promote the physiological haemostatic effect of uterine muscle retraction, rather than a balloon. STUT is an off‐label method used to achieve this mechanism where the Jada System and other purpose‐built vacuum‐induced haemorrhage control devices remain unavailable. Alternative re‐usable purpose‐built alternatives are also available in India [27].

Off‐label STUT has been achieved with devices including a large steel uterine suction curette [35], a re‐purposed Bakri balloon [36], a Foley catheter [37], and simple flexible plastic catheters (e.g., Levin stomach tube) [38, 39, 40]. Examples of the various suction uterine tamponade devices are shown in Table S1.

Other purpose‐built PPH suction devices and drains on the horizon include those by Koko Medical (https://www.kokomed.com/), Youdim Pharmaceutics (https://www.youdim.com/resqmedical), and Raydiant Oximetry (https://raydiantoximetry.com/technology). Other off‐label (‘repurposed’) devices for PPH have been documented by Impact Global Health (https://www.impactglobalhealth.org/data/maternal‐health‐pipeline/dashboard).

Historically, robust evidence for the effectiveness of interventions for refractory PPH has been strikingly deficient [21]. Observational studies have reported conflicting results [41, 42]. Combined results of two small randomised trials suggest greater effectiveness and less pain with STUT than UBT [27, 43]. In this meta‐analysis, death or blood loss > 1000 mL was less frequent with STUT than UBT (risk ratio 0.56, 95% confidence interval 0.33–0.97) [43]. While available data support the effectiveness of STUT, far larger studies are needed to confirm safety. The WHO‐sponsored RED trial is one such trial [44].

## 5 The WHO Pathway for Equitable Access to Medical Devices

Compared with coordinated industry processes, the use of improvised or off‐label devices often follows a stuttering, imperfect adoption pathway, largely driven by enthusiasts who champion the device. Industry cannot afford to invest in research on low‐cost, reusable novel devices on which they will not get their money back in sales. Although philanthropists, charities, and governmental aid programmes attempt to fill some of these gaps, the development of any individual technology may be uncoordinated and sporadic, leading to devices with an incomplete evidence base.

To address this problem, there are various processes developed by the WHO to assist in global access to essential public health products.

The product development process begins with the identification of an unmet public health need. The development process utilizes either a preferred product characteristic (PPC) approach for products in the preclinical phase or a target product profile (TPP) for products requiring Phase 3 clinical trials. These serve as guidance throughout the development process.

WHO also has a committee that provides ‘Coordinated Scientific Advice’ (CSA) [45]. Through this, product developers may approach WHO and obtain advice on the most appropriate way to generate high‐quality evidence on a product's benefits and risks with the intention of obtaining a WHO policy recommendation or obtaining prequalified status if the product is found to meet WHO requirements.

Health products are rigorously assessed to be included in WHO Guidelines, using the GRADE process to assess clinical efficacy of the device, and also acceptability, feasibility, cost effectiveness, resource requirements, stakeholder preferences and effect on health equity. National regulatory committees in LMICs rely on this robust WHO assessment to provide clarity and advice as to which devices and medicines to adopt and use in country. WHO approval through its guideline approval process therefore provides an important route to making devices accessible, affordable and adopted worldwide.

If this can be achieved, then it opens the door to approval on the WHO Compendium of Innovative Health Technologies for low‐resource settings, a device approval process equivalent to the WHO Essential Medicines List [46]. An important addition would be a formal ‘post‐recommendation survey’ analogous to commercial post‐marketing surveys, essential for picking up signals of possible unanticipated harms too infrequent to be identified in randomised trials.

## 6 Conclusions

The development and distribution of maternal health devices is critical for improved maternity care worldwide. Novel devices that address critical public health priorities can benefit from WHO pathways that assist in bringing essential low‐cost products to a global market. Improvisation and off‐label use of affordable, widely available devices can also massively increase global access to new technologies, but pathways are needed to ensure that these receive the same attention and safeguards as commercial products.

Global agencies and professional organisations can play a facilitatory role by rapidly evaluating evolving evidence and updating clinical practice recommendations and providing co‐ordination of research efforts, platforms for data sharing, and instructions for use on off‐label devices. This will support the use of effective interventions and provide clinical guidance on their safe and appropriate use.

**Table:** 

Future research Systematic review of current evidence on improvised devices is needed to inform global and national guidelines Robust randomised trials are required of promising off‐label devices such as suction tube uterine tamponade, uterine tourniquet, and transvaginal clamping of the uterine arteries for postpartum haemorrhage Creation of a centralised agency to collate ongoing evidence and provide advice and protocols for the use of off‐label and improvised devices would assist with standardisation and safety

## Author Contributions

G.J.H. has the original idea for the paper and wrote the first draft. This was edited and added to by M.S.‐M., S.D.R. and A.D.W. All authors approved the final text.

## Conflicts of Interest

G.J.H. conceived the off‐label use of suction tube uterine tamponade (STUT) and the MaternaWell Tray, and contributed to the conception of the free‐flow glove balloon and the Ellavi free‐flow balloon. He has no financial interest in these devices. A.D.W. developed the BabySaver tray for neonatal resuscitation with design engineer Peter Watt at the University of Liverpool. The sales rights for Africa were sold to the Sanyu Africa Research Organisation for £1 in 2019, and he has no financial interest in the device. He has no other conflicts of interest. S.D.R. contributed to the Jada System prior to acquisition by Organon and to the MaternaWell Tray at Equalise Health. She has no financial interest in these devices.

## Supporting information


**Table S1:** Comparison of a selection of suction uterine tamponade devices.

## Data Availability

Data sharing not applicable to this article as no datasets were generated or analyzed during the current study.
