# Supplementary material for: Achieving Equitable Access to Obstetric Devices Through Innovation, Improvisation and Off‐Label Use
Source: BJOG. 2025 Oct 14;132(13):1903–9. doi: 10.1111/1471-0528.70058 (PMC12592759; doi:10.1111/1471-0528.70058)
Supplement: Supplementary file 1 — Table S1: Comparison of a selection of suction uterine tamponade devices. [file BJO-132-1903-s001.docx]

Supplementary Table 1. Comparison of a selection of suction uterine tamponade devices.

|  | Purpose-designed | | Off-label use | |
| --- | --- | --- | --- | --- |
|  | Jada* | Panicker | Bakri / Foley catheter | Suction tube e.g. Levin |
| Availability | Limited (U.S. & Canada) | Limited (India) | Variable | Widespread |
| Cost (approx.) | USD 1000^[[1]](#endnote-1)^ | Re-usable | Variable | USD 0.50c |
| Use with undilated cervix post caesarean | No | Yes | Yes | Yes |
| Additional insertion step to inflate cuff | Yes | No | Yes | No |
| Comparative data versus balloon | Observational | RCT (small) | No | RCT (small) |

1. . Lee D, Stuart S, & Franco-Marx Y. Intrauterine vacuum-induced hemorrhage-control device for hemorrhage after myomectomy: a case report and review of the literature. Gynecology And Pelvic Medicine 2024;7. [↑](#endnote-ref-1)
